# Visual context processing in bipolar disorder: a comparison with schizophrenia

**DOI:** 10.3389/fpsyg.2013.00569

**Published:** 2013-08-30

**Authors:** Eunice Yang, Duje Tadin, Davis M. Glasser, Sang Wook Hong, Randolph Blake, Sohee Park

**Affiliations:** ^1^Department of Psychology, Vanderbilt University, NashvilleTN, USA; ^2^School of Optometry, University of California Berkeley, BerkeleyCA, USA; ^3^Center for Visual Science and Department of Brain and Cognitive Sciences, University of Rochester, RochesterNY, USA; ^4^Department of Ophthalmology, University of Rochester, RochesterNY, USA; ^5^Department of Psychology, Florida Atlantic UniversityBoca Raton, FL, USA; ^6^Department of Brain and Cognitive Sciences, Seoul National UniversitySeoul, Republic of Korea

**Keywords:** bipolar disorder, contextual effects, perception deficit, visual processing, schizophrenia

## Abstract

Anomalous perception has been investigated extensively in schizophrenia, but it is unclear whether these impairments are specific to schizophrenia or extend to other psychotic disorders. Recent studies of visual context processing in schizophrenia (Tibber et al., 2013; Yang et al., 2013) point to circumscribed, task-specific abnormalities. Here we examined visual contextual processing across a comprehensive set of visual tasks in individuals with bipolar disorder and compared their performance with that of our previously published results from schizophrenia and healthy participants tested on those same tasks. We quantified the degree to which the surrounding visual context alters a center stimulus' appearance for brightness, size, contrast, orientation and motion. Across these tasks, healthy participants showed robust contextual effects, as indicated by pronounced misperceptions of the center stimuli. Participants with bipolar disorder showed contextual effects similar in magnitude to those found in healthy participants on all tasks. This result differs from what we found in schizophrenia participants (Yang et al., 2013) who showed weakened contextual modulations of contrast but intact contextual modulations of perceived luminance and size. Yet in schizophrenia participants, the magnitude of the contrast illusion did not correlate with symptom measures. Performance on the contrast task by the bipolar disorder group also could not be distinguished from that of the schizophrenia group, and this may be attributed to the result that bipolar patients who presented with greater manic symptoms showed weaker contrast modulation. Thus, contrast gain control may be modulated by clinical state in bipolar disorder. Stronger motion and orientation context effects correlated with worse clinical symptoms across both patient groups and especially in schizophrenia participants. These results highlight the complexity of visual context processing in schizophrenia and bipolar disorder.

## Introduction

Visual dysfunction represents a core dimension of schizophrenia, but its role in the etiology of the disease has yet to be defined. To address this shortcoming, recent studies have investigated a number of visual functions as potential biomarkers for the disease, with contextual processing being one of those candidates (Carter and Barch, 2007; Gold et al., 2012). Contextual processing serves to enhance differences among visual features and consequently facilitate their segmentation from their background (Albright and Stoner, 2002). As a result, the perceptual appearance of a visual feature is altered in such a way as to emphasize its relative difference from features in the surrounding spatial context. Recent studies suggest that individuals with schizophrenia (SZ) are less influenced by visual context on some tasks, thereby enabling them to perceive the absolute characteristics of visual features more accurately (e.g., Tadin et al., 2006; Uhlhaas et al., 2006). For example, in the center-surround contrast illusion presence of a high-contrast background decreases the apparent contrast of smaller foreground features. Several studies have reported more accurate performance at judging stimulus contrast in SZ relative to controls, which implicates a weakened contextual effect of contrast (Dakin et al., 2005; Barch et al., 2012; Tibber et al., 2013). Considered together, these results seem to suggest existence of a generalized contextual processing deficit in schizophrenia. However, we recently showed that this deficit in contextual processing does not generalize across all contextual cues when explored within the same group of SZ: the magnitude of contextual modulations of luminance, size, orientation, and motion, were comparable to those of healthy controls, despite a weakened contextual effect of contrast (Yang et al., 2013). In addition, the strength of certain contextual illusions (i.e., orientation and motion repulsion) was predictive of symptom severity and social functioning in SZ. Thus, impairments in contextual processing in schizophrenia may not be as wide-ranging as previously thought, and in those visual sub-modalities where impairment is evident the degree of impairment may be modulated by illness severity.

Knowing the diagnostic specificity of putative contextual processing abnormalities in schizophrenia is as important as understanding the conditions under which contextual deficits arise. It may be that contextual disturbances are related more broadly to psychosis rather than just the phenotype of schizophrenia. One approach for addressing this issue is to investigate contextual processes in individuals with other forms of psychosis, for example bipolar disorder. In one study, the surround contrast illusion was examined in individuals with bipolar disorder (BD), but they were a part of heterogeneous clinical “control” group, which consisted of individuals with affective, personality, and post-traumatic stress disorders (Dakin et al., 2005). Thus, while the clinical control group showed no contextual deficits, there was no information specific to bipolar disorder. Investigating contextual effects in bipolar disorder may also speak to the issue of whether schizophrenia and bipolar disorder occupy different regions of a continuum or are distinct disorders. They share similar symptoms such as hallucinations and delusions, are often treated with identical antipsychotic medications, and may share some genetic liability (Purcell et al., 2009; Van Snellenberg and De Candia, 2009). Some commonly reported visual deficits in schizophrenia are also found in bipolar disorder, including impairments in visual backward masking (Green et al., 1994; review by McClure, 1999), in vernier acuity (Kéri et al., 2004, 2007), and in early sensory processing measured with visually evoked potentials (Yeap et al., 2009). However, it must be noted that SZ and BD are distinguished by their performance on a very broad range of tasks from perceptual/cognitive to motor domains. In the visual perceptual domain, SZ and BD patients perform differently on tasks measuring photoreceptor sensitivity (Balogh et al., 2008), motion discrimination (Chen et al., 2006), and notably, contrast sensitivity modulation by collinear flanking stimuli (Kéri et al., 2005).

Given the current state of the literature, one cannot say whether the two disorders are distinct or fall on a continuum. Examining visual function in bipolar disorder may provide evidence for the specificity of contextual disturbances in schizophrenia if the two groups show distinct patterns of deficits and intact functions. This information, in turn, might be important to determine biomarkers specific to schizophrenia. The current study aims to systematically explore contextual processing in bipolar disorder in order to determine the extent to which contextual abnormalities are shared or specific to schizophrenia. We employed the same contextual tasks in BD as those used in our previous study of SZ and healthy controls (CO) and compared the pattern of contextual modulation in bipolar disorder to our previously published results (Yang et al., 2013).

## Materials and methods

### Participants

Sixteen individuals who met the DSM-IV (Diagnostic and Statistical Manual of Mental Disorders, fourth edition) criteria for bipolar disorder were recruited from Nashville, Tennessee. Diagnosis was confirmed by trained master's- and doctoral-level psychologists using the Structured Clinical Interview for the DSM-IV (First and Gibbon, 1997). Excluded from the study were individuals who reported any substance use within the last 6 months, and individuals with a history of neurological disorders or head trauma, or an IQ lower than 80 on the National Adult Reading Test (NART; Nelson, 1982). All participants had visual acuity of 20/30 or better (Optec Vision Tester 5000, Stereo Optical, Chicago, IL), with refractive correction if needed using a kit of trial lenses. Behavioral and clinical data of BD were compared with those of SZ (*N* = 30) and CO (*N* = 23) reported in our previous study (Yang et al., 2013).

Table 1 summarizes the demographic information for the BD group tested in this study together with the SZ and CO individuals tested in our earlier work. The mean illness duration of BD was significantly briefer than the duration of illness in SZ [*t*_(44)_ = 2.4, *p* = 0.02]. All but 2 BD were medicated (79% on atypical antipsychotic drugs, 86% on mood stabilizers, and 64% taking both). The mean chlorpromazine equivalent dose (CPZ) was significantly higher in SZ than in BD at the time of testing [*t*_(34)_ = 3.1, *p* = 0.004]. Clinical symptoms in both patient groups were assessed with the Brief Psychiatric Rating Scale (BPRS; Overall and Gorham, 1962) and SZ and BD showed comparable BPRS scores (*p* > 0.05). BD were also rated on the Young Mania Rating Scale (YMRS; Young et al., 2000) and the Hamilton Rating Scale of Depression (HRSD; Hamilton, 1960). SZ were rated on the Scale of Assessment for Positive and Negative Symptoms or SAPS and SANS, respectively (Andreasen, 1983, 1984). Both patient groups were clinically stable at the time of testing, as assessed by the ratings scales mentioned above and by self-reports of episodes or hospital admittance in the last 6 months.

**Table 1:** **Demographic and clinical information on subject groups**.

	**BD**	**SZ**	**CO**
**DEMOGRAPHIC INFORMATION**			
N	16	30	23
Mean age	34 (10)	41 (8)	39 (9)
Gender (M/F)	7/9	11/19	11/12
Mean NART IQ	109 (10)	104 (9)	106 (11)
Social functioning^*^	115 (7)	111 (9)	123 (5)
**CLINICAL CHARACTERISTICS**			
Mean illness duration (years)^*^	11 (8)	17 (8)	
CPZ equivalent (mg/kg/day)^*^	224 (167)	496 (365)	
BPRS	11 (8)	13 (8)	
SAPS	–	14 (13)	
SANS	–	17 (7)	
YMRS	7 (8)	–	
HRSD	10 (6)	–	

Asterisks indicate significant group differences (p < 0.05). Parentheses denote standard deviation (SD). NART, national adult reading test; CPZ, chlorpromazine daily equivalent; BPRS, brief psychiatric rating scale; SAPS and SANS, scale of assessment for positive and negative symptoms, respectively; YMRS, young mania rating scale; HRSD, Hamilton rating scale of depression.

There were no significant differences in mean age, mean NART IQ, and in the proportion of women among all three groups (all *p* > 0.05). Social functioning, as assessed with the Social Functioning Scale (Birchwood et al., 1990) was worse in both patient groups relative to CO [*F*_(2, 61)_ = 13.2, *p* < 0.001; BD vs. CO: *t*_(34)_ = 4.0, *p* < 0.001; SZ vs. CO: *t*_(43)_ = 5.4, *p* < 0.001]. The Institutional Review Board of Vanderbilt University approved this study protocol. All participants provided written informed consent and were paid.

### Apparatus

The study design was identical to that of Yang et al. (2013). Stimuli were created in MATLAB and the Psychophysics Toolbox (Brainard, 1997; Pelli, 1997) and were presented on a linearized CRT monitor (1280 × 960 resolution; 120 Hz). Viewing distance was 73 cm. Head position was stabilized by a chin rest. The display background was gray (luminance = 35.2 cd/m^2^, except in the brightness induction task, where luminance was 0.11 cd/m^2^). The ambient illumination was 0.16 cd/m^2^.

### Context battery

**Figure 1:**
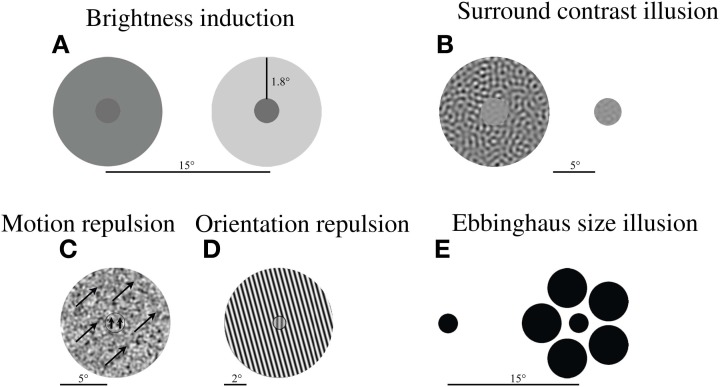
**Example stimuli in each experiment. (A)** Brightness induction illusion: The reference stimulus (left) with darker surround appears brighter that the target stimulus (right) of equal luminance with lighter surround. **(B)** Surround contrast illusion: A stimulus with a high contrast pattern in the surround (left) appears weaker in contrast relative to the reference stimulus (right) of equal contrast. **(C)** Motion and **(D)** orientation repulsion: The motion direction or orientation of a center stimulus appears to be repelled away from the motion direction or orientation of the surrounding pattern (arrows denote motion direction). **(E)** Ebbinghaus size illusion: A circle appears smaller when presented with large circles in the surround (right), relative to a stimulus of equal size (left). Note: Scale bar denotes the stimulus display size in degrees of visual angle. The spacing between stimuli in 1A is not on the same scale as the size of the stimuli.

To assess contextual effects in a broad range of stimulus dimensions (luminance, contrast, size, orientation, and motion direction), we developed a battery of five psychophysical tasks (Yang et al., 2013). All tasks involved a center stimulus (Figure 1), whose perceptual appearance was altered by the presence of surrounding stimuli. In these tasks, participants were instructed to judge the appearance of the center stimulus by comparing it with a fixed reference stimulus (luminance, size, and contrast tasks) or by judging its deviation from vertical (motion and orientation tasks). To quantify the magnitude of the contextual modulation, the point of subjective equality (PSE) was measured for each task (as described below). PSEs were estimated by adaptive staircases for all tasks except the brightness induction task, where the method of adjustment was used. Stimuli were always presented until a response was made, except for the motion task, where stimulus duration was fixed at 200 ms. To establish baseline performance and to ensure that participants accurately judged stimulus dimensions tested in different tasks, all tasks included a no-context control condition. This condition was identical to the main context condition except that no surrounding context was present.

### Brightness induction task

The stimulus consisted of two circles (0.5° radius) surrounded by annuli (2.4° radius). They were simultaneously presented 15° apart (Figure 1A). The luminance of the reference circle (always shown on the left) was fixed at 6 cd/m^2^, while its surrounding annulus was set to 8, 12, 16, 20, or 24 cd/m^2^. A range of surround luminance values was included to allow comparison of the pattern of surround modulation between groups. The initial luminance of the target stimulus (always shown on the right) was randomly chosen from a range of 2–14 cd/m^2^, while the luminance of its annulus was fixed at 24 cd/m^2^. Fixed stimulus positions were used to control spatial inhomogeneities in screen luminance. Participants' task was to adjust the luminance of the target circle on the right to match the luminance of the reference circle on the left. By pressing one of two keys, participants adjusted the target luminance, decreasing or increasing luminance in steps of 0.2 cd/m^2^. Three such adjustments were performed for each surround luminance, with their average taken as the PSE. The strength of brightness induction was defined as the difference (in cd/m^2^) between fixed luminance of the reference circle (6 cd/m^2^) and the perceived (i.e., adjusted) luminance of the target (which was typically much higher).

### Surround contrast illusion task

The stimulus display (Figure 1B) was similar to surround contrast illusion stimuli used in previous studies (Chubb et al., 1989; Dakin et al., 2005; Barch et al., 2012). The display consisted of two circular patches (1.67° radius; 13.5° horizontal center-center separation). Each patch was filled with spatial frequency filtered noise (1 cycle/° center frequency; 0.25-octave bandwidth). The Michelson contrast of the reference patch was fixed at 20%, while the surrounding high-contrast noise annulus (6.67° radius) was shown at 97% contrast. The starting contrast of the target stimulus was randomly chosen (10–30%). On each trial, the positions of reference and target stimuli were randomly assigned, and participants judged which patch appeared higher in contrast by a key press. These responses were used to adaptively adjust the contrast of the target stimulus to match the apparent contrast of the reference stimulus.

### Surround motion repulsion task

The display (Figure 1C) consisted of a stimulus moving within a small circular aperture (1° radius) surrounded by another stimulus moving within a large annulus (6° radius). Stimulus speed for both the center and the annulus was 3°/s. The stimuli were composed of spatial frequency filtered noise (80% contrast; 1°/degree center frequency; 0.25-octave bandwidth). The direction of surround motion was either 45° clockwise or 45° counterclockwise from vertical. The direction of the center motion was either 18° clockwise or 18° counterclockwise at the start of the task and thereafter was varied by the staircase procedure. The stimuli were presented for 200 ms and, then, were immediately replaced with a blank screen. This was done to avoid pursuit eye movements. Participants' task was to indicate by a key press whether the central motion direction was clockwise or counterclockwise relative to vertical.

### Surround orientation repulsion task

The display (Figure 1D) consisted of a small circular grating (0.5° radius, 50% contrast, 3 cycles/°) surrounded by a large, high-contrast annulus (4° radius, 97% contrast, 3 cycles/°). The phase of each grating was random. The orientation of the annulus was always 15° counterclockwise from vertical. At the start of the task the center orientation was either 11° clockwise or counterclockwise and thereafter determined by the staircase procedure. Participants' task was to judge whether the center patch appeared tilted clockwise or counterclockwise relative to vertical and to indicate their responses by a key press.

### Ebbinghaus size illusion task

This task was a variant of the classic Ebbinghaus illusion (Figure 1E). The display consisted of the target and the reference stimuli presented 15° apart (center-center). Their positions (left or right) were randomly assigned on each trial. The fixed reference stimulus consisted of a small dark circle (1.08° radius) surrounded by five evenly spaced large circles (2.17° radius and a 4° center-to-center distance from the reference stimulus). The target stimulus was a small circle. Its initial radius was randomly chosen between 0.92° and 1.08°, and thereafter varied by the staircase procedure (described below). All stimuli were presented at 97% contrast. Participants' task was to judge which of the two center circles was larger and to indicate their responses by a key press.

### Procedure

The order of tasks was randomized for each participant. The experiment for each task consisted of four blocks, starting with the no-context control block and followed by three context blocks. In each block, two interleaved one-up/one-down staircases were used to estimate PSEs. The step size of these staircases decreased after every two reversals. The staircases converged after seven reversals. For each staircase, the PSE estimate was based on the average of the last four reversals. The resultant PSE for each participant was an average of six such staircases (two staircases in each of three blocks). For control tasks, PSEs were based on the average of two staircases. One exception was the brightness induction task, where the above-described adjustment method was used. No feedback was provided and there was no time limit for making a response. The entire context battery took about 1–1.5 h to complete. Before starting each task, participants were given detailed instructions. Each task started with five practice trials.

The strength of contextual effects was measured by quantifying the change in PSE values measured in the presence of a surrounding context relative to PSE values measured in the control condition with no surrounding context (i.e., as the degree to which a participant's perception changed after adding the surrounding context). The measurement units for luminance, contrast, and size tasks were cd/m^2^, log_10_ contrast, and arcmin, respectively. Orientation and motion angular repulsions were measured in degrees.

### Psychometric properties

We considered the following psychometric issues: ceiling effects, floor effects, and measurement reliability (Table 2). All tasks had inherent stimulus-constrained ceilings (e.g., 90° repulsion in the orientation task). All results were considerably weaker than these ceilings. Floor effects would be manifested as a “no contextual effect” for each task. However, because CO participants exhibited strong contextual effects, we had ample dynamic ranges to reveal potential weakening of contextual processing in clinical groups. Finally, we found no deviation from normality and equality of variance, as assessed with Kolmogorov-Smirnov test and Levene's test, respectively.

**Table 2:** **Split-half reliability scores for each task and for each group**.

	**BD**	**SZ**	**CO**
Orientation	0.86	0.89	0.58
Size	0.74	0.90	0.90
Motion	0.89	0.78	0.73
Contrast	0.70	0.83	0.84
Luminance	0.89	0.88	0.87

To examine measurement reliability, we split each data set into halves or thirds and correlated these partial data sets. Table 2 depicts split-half reliability scores for each task and for each group. For size and contrast tasks, where we obtained six independent PSE estimates, we split the data into halves. For motion and orientation tasks, we obtained three pairs of measurements, where each pair consisted of two center directions/orientations. To assess measurement reliability, we correlated the second and third estimates. The modest correlation for CO (*r* = 0.58) in the orientation task is largely due to a single CO participant who failed to show a contextual effect on one measurement; without that individual's data, the reliability is 0.71. Note that somewhat lower numbers in motion and orientation tasks are expected, given that only two thirds of the data are used to compute reliabilities. Finally, second and third adjustment estimates in the brightness task were correlated. In sum, we found reliabilities for BD, SZ, and CO to be comparable for each task and relatively high. For BD, all split-data reliabilities were between 0.70 and 0.89 (average = 0.78).

### Analysis

For all tasks except the brightness induction task, univariate analysis of variance (ANOVA) was used to compare performance measures of BD, SZ, and CO. In the brightness induction task, repeated measures analysis of variance (ANOVA) compared performance between the three groups with five surround luminance conditions as the within-subjects factor. *Post-hoc* comparisons were performed using Welch's *t*-test. Effects sizes were reported for ANOVAs and *t*-tests using partial η^2^ and Cohen's *d*, respectively. Below, we compare performance measures of BD with those of SZ and CO for each task. This is followed by combined analyses across tasks that compare the three groups using mixed model ANOVA. Pearson's *r* was used to determine correlations among contextual tasks, and Spearman's *rho* (*r*_*s*_) was used to test for correlations among contextual effects and clinical measures. We have reported both raw *p*-values and alpha levels adjusted for Bonferroni correction of multiple comparisons.

## Results

Owing to experimenter error, data for contrast and brightness tasks were missing for 1 BD participant. However, data on the other tasks were retained. Moreover, if any participant's data fell three standard deviations or more from the group mean, his or her baseline and context data were excluded for that task. This resulted in the exclusion of five data sets (three for motion and two for contrast), accounting for approximately 3% of all data. Two of the three outliers for the motion task were control participants. The two outliers in the contrast task were bipolar participants, one being the same outlier as in the motion task. ANOVAs revealed that BD, SZ, and CO did not significantly differ in each of the baseline conditions in which surround stimuli were absent (all *p* > 0.1). This result shows that patient groups had no problems accurately performing the visual tasks used in this study.

**Figure 2:**
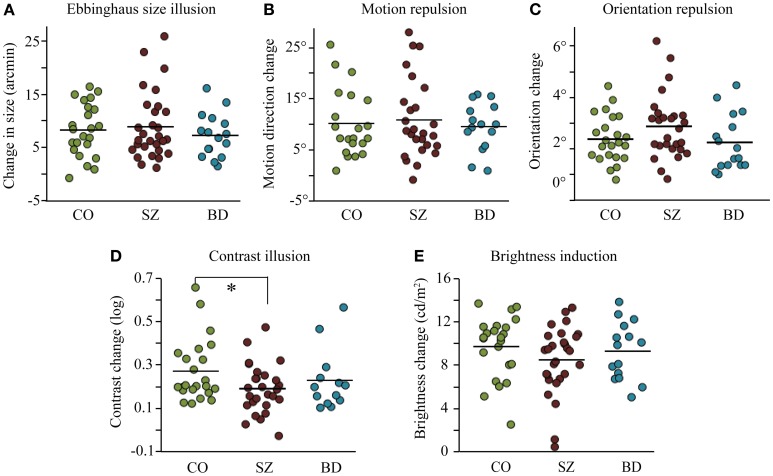
**Results from five context modulation experiments in individuals with schizophrenia (SZ), individuals with bipolar disorder (BD), and participants in the control group (CO): (A) Ebbinghaus size illusion, (B) motion repulsion, (C) orientation repulsion, (D) surround contrast illusion, and (E) brightness induction illusion (the condition with the strongest surround modulation (8 cd/m^2^) is represented).** Data points represent individuals within each group and bars denote mean group performance. The only significant group difference (^*^) was weaker contextual modulation of contrast in SZ relative to CO [*t*_(42)_ = 4.87, *p* = 0.03, *d* = 0.64].

Surround contextual effects were observed across all tasks for each group (one-sample *t* tests, all *p* ≤ 0.001, adjusted α = 0.01, reflecting 5 comparisons per group, Figure 2). In the brightness induction task, we found a main effect of surround luminance [*F*_(4, 242)_ = 423.0, *p* < 0.001, partial η^2^ = 0.87], but no main effect of group [*F*_(2, 63)_ = 0.77, *p* = 0.47, partial η^2^ = 0.02] and no interaction between luminance and group [*F*_(8, 252)_ = 1.15, *p* = 0.33, partial η^2^ = 0.04]. We also compared group performance on the surround luminance condition that would evoke strongest illusion (surround luminance of 8 cd/m^2^) and found no significant difference across groups (Table 3; Figure 2E). The contextual effects of size, motion, and orientation, were not significantly different across the three groups (Table 3; Figures 2A–C). In the contrast task, the group difference reached significance only at a single-tailed unadjusted alpha level (*p* = 0.096, α = 0.05). Our previous study showed weakened contextual modulation of contrast in SZ relative to CO [*t*_(42)_ = 4.87, *p* = 0.03, *d* = 0.64]. For the purposes of our study, we thought it was worth employing paired *t*-tests to determine whether the contextual contrast effect in BD was similar in magnitude to that of SZ and to CO. Indeed, BD was not significantly different from either CO [*t*_(25)_ = 0.76, *p* = 0.39, *d* = 0.29] or SZ [*t*_(20)_ = 0.75, *p* = 0.4, *d* = 0.33; Figure 2D] in the contrast illusion.

**Table 3:** **Results of ANOVAs comparing contextual effects of schizophrenia, bipolar, and control groups in each task**.

**Task**	***F***	***df***	***p***	**Partial η^2^**
Orientation	1.96	63	0.150	0.058
Size	0.48	65	0.621	0.015
Motion	0.19	59	0.824	0.007
Contrast	2.43	60	0.096	0.075
Luminance	1.15	63	0.323	0.035

Adjusted alpha level = 0.01, reflecting correction for 5 multiple comparisons.

**Figure 3:**
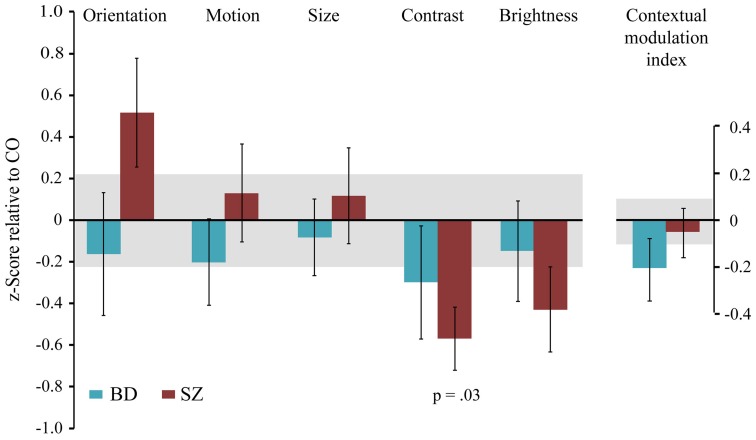
**The magnitude of contextual modulation in BD and SZ.** The magnitude of contextual effect in individuals with bipolar disorder (BD) and individuals with schizophrenia (SZ) was converted into *z* scores for each task relative to the respective mean and variance of the control group. The contextual modulation index represents the average *z* score across tasks for each participant. Negative values indicate weaker contextual modulation in patients, whereas positive values indicate stronger contextual modulation in patients relative to the control group. As noted in Figure 2, SZ exhibited a significantly weaker contrast illusion compared to CO. Error bars denote the standard error of the mean of the *z* scores in clinical groups, and the shaded region denotes the standard error of the mean of the control group.

To examine the pattern of results across all contextual tasks, we normalized effect sizes for each task relative to the performance of CO to derive z scores (Figure 3). For the brightness induction task, we used the PSE estimate in the surround luminance condition that would evoke the strongest illusion (surround luminance of 8 cd/m^2^). Using a mixed model ANOVA with task (5) and group (3) as fixed factors, we found no significant main effect of group, *F*_(2, 65)_ = 0.54, *p* = 0.59; or task, *F*_(4, 64)_ = 1.6, *p* = 0.18; nor a significant interaction between group and task, *F*_(8, 64)_ = 1.5, *p* = 0.16.

To estimate a general measure of contextual processing, we derived a contextual modulation index (CMI) for each patient by averaging *z* scores across tasks (relative to CO). If BD showed a general weakening of contextual processing, then CMI should be negative. A positive CMI would indicate a general strengthening of contextual processing. The result for BD, however, is a *z* value of −0.2, with an associated *p*-value of 0.84 (Figure 3). In other words, CMI is nearly zero for BD, as was the CMI for SZ (*z* = −0.048, *p* = 0.96). Furthermore, variance did not differ between groups [Levene's test: *F*_(2, 66)_ = 0.24, *p* = 0.79], ruling out the possibility that the absence of CMI differences is due to equal numbers of BD with abnormally strong and abnormally weak CMIs.

We also examined intertask correlations to test whether a weak contextual effect on one task would predict a weak contextual effect on other tasks. However, no significant correlations were found within any of the three groups (Table 4). It should be noted that some intertask correlations were trending toward significance at an unadjusted alpha level (0.05) but the relationship differed in each group: orientation and motion were modestly correlated in SZ (*r* = 0.39, *p* = 0.07), size and motion in BD (*r* = 0.5, *p* = 0.06), luminance and motion in CO (*r* = 0.4, *p* = 0.07). However, there was no consistent trend of intertask correlation across groups. It is worth noting that these low correlations are not caused by low measurement reliability, as split-half reliabilities were high (Table 2).

**Table 4:** **Intertask correlations within and across groups**.

		**Size**		**Motion**		**Contrast**		**Luminance**	
		***r***	***p***	***r***	***p***	***r***	***p***	***r***	***p***
Orientation	*BD*	0.17	0.52	−0.12	0.66	−0.10	0.76	0.17	0.54
	*SZ*	−0.15	0.48	0.39	0.07	0.02	0.94	0.04	0.86
	*CO*	0.21	0.34	0.09	0.71	0.22	0.31	0.06	0.79
	*All*	0.03	0.79	0.24	0.07	0	1	0.04	0.77
Size	*BD*			0.5	0.06	−0.11	0.72	0.32	0.25
	*SZ*			−0.16	0.45	−0.17	0.39	−0.04	0.83
	*CO*			−0.21	0.35	−0.14	0.52	0.08	0.73
	*All*			−0.08	0.54	−0.14	0.27	0.04	0.78
Motion	*BD*					0.41	0.17	0.12	0.68
	*SZ*					0.02	0.71	0.14	0.49
	*CO*					0.14	0.54	0.40	0.07
	*All*					0.14	0.31	0.2	0.12
Contrast	*BD*							0.05	0.86
	*SZ*							0.02	0.93
	*CO*							−0.22	0.31
	*All*							−0.01	0.94

Adjusted alpha level = 0.005, reflecting correction for 10 multiple comparisons per group. r, Pearson's correlation coefficient; p, significance level.

**Figure 4:**
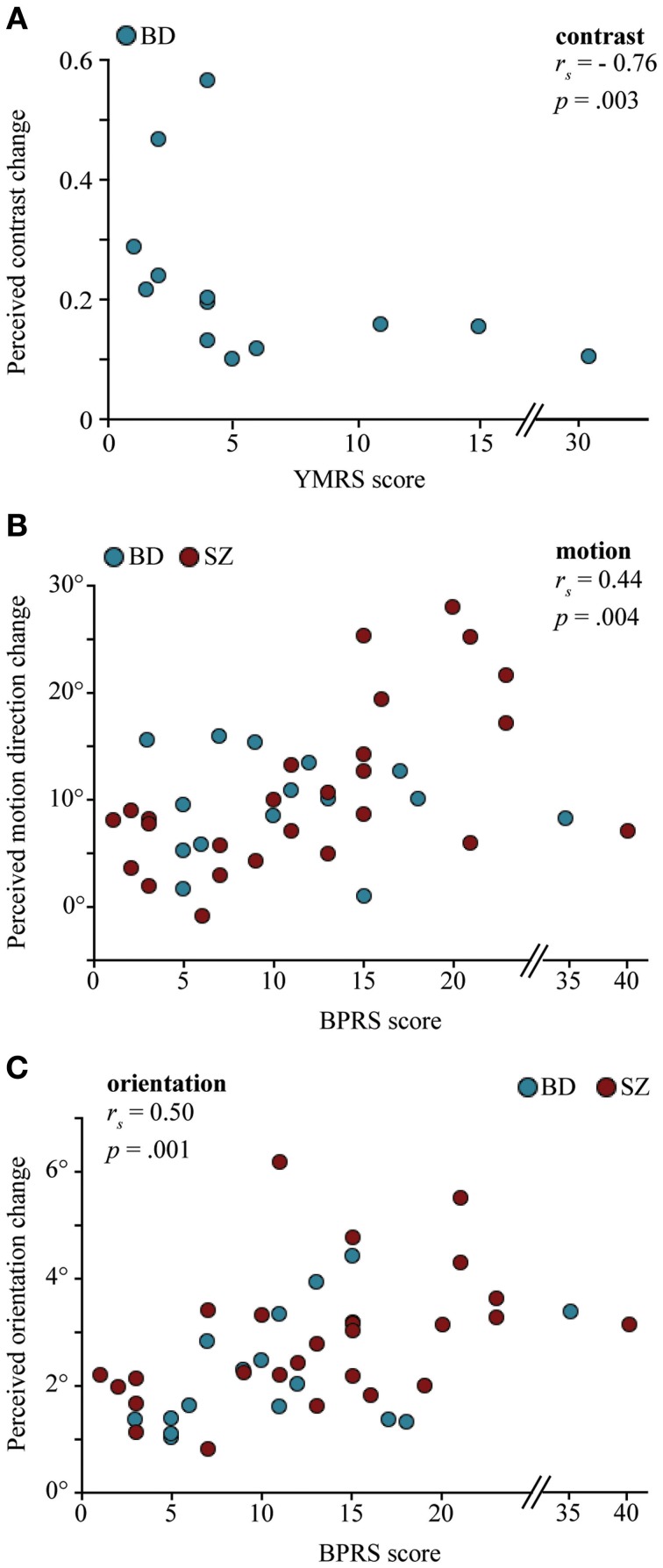
**The relationship between the magnitude of contextual effects and clinical measures in individuals with bipolar disorder (BD) and individuals with schizophrenia (SZ). (A)** Correlation between the Young Mania Rating Scale (YMRS) symptoms ratings and perceived contrast changes in the surround contrast illusion in BD: Higher mania scores were associated with weaker contrast illusion. **(B** and **C)** Correlations between Brief Psychiatric Rating Scale (BPRS) symptoms ratings and perceived motion direction changes in the motion repulsion task and perceived orientation changes in the orientation repulsion task. Patients with higher BPRS scores were more likely to exhibit stronger repulsion effects. Correlations remained significant when excluding three potential YMRS outliers in (A), *r*_*s*_ = −0.68, *p* = 0.03, and 2 potential BPRS outliers in **(B)** and **(C)**, *r*_*s*_ = 0.53, *p* = 0.001; *r*_*s*_ = 0.47, *p* = 0.002, respectively.

Finally, we examined the relationships between the strength of contextual illusions and clinical measures in patient groups (Table 5). Unless otherwise noted, significance was defined at an adjusted alpha level of 0.01, reflecting Bonferroni correction for five multiple comparisons per clinical measure. In BD, the contextual modulation of contrast negatively correlated with YMRS score (Figure 4A): greater manic symptoms were associated with a weaker surround contrast illusion (*r*_s_ = −0.76, *p* = 0.003). When excluding three potential outliers based on YMRS score (see Figure 4A), the correlation remained significant at the unadjusted alpha level (*r*_s_ = −0.68, *p* = 0.03, α = 0.05). There was also a trend for the severity of depressive symptoms (HRSD) to positively correlate with the magnitude of orientation repulsion illusion in BD (*r*_*s*_ = 0.45, *p* = 0.08). Similarly in SZ, the strength of the orientation illusion was associated with greater positive symptoms (SAPS; *r*_*s*_ = 0.38, *p* = 0.05) and negative symptoms (SANS; *r*_*s*_ = 0.46, *p* = 0.02). However, these correlations in SZ did not survive Bonferroni correction.

**Table 5:** **Correlations between context measures and clinical and demographic variables**.

		**Orientation**		**Size**		**Motion**		**Contrast**		**Luminance**	
		***r***	***p***	***r***	***p***	***R***	***p***	***r***	***p***	***r***	***p***
BD	YMRS	−0.06	0.84	−0.15	0.58	−0.44	0.1	−0.76	0.003^**^	−0.12	0.67
	HRSD	0.45	0.08	−0.27	0.31	−0.25	0.38	0.44	0.14	0.22	0.43
	BPRS	0.48	0.06	−0.25	0.35	−0.03	0.93	0.07	0.83	0.19	0.49
SZ	SAPS	0.38	0.05^*^	−0.02	0.9	0.44	0.02^*^	0.12	0.56	0.32	0.1
	SANS	0.46	0.02^*^	−0.02	0.9	0.41	0.04^*^	0.13	0.52	0.28	0.16
	BPRS	0.50	0.008^**^	−0.04	0.84	0.57	0.002^**^	0.16	0.42	0.20	0.30
Patients	BPRS	0.50	0.001^**^	−0.10	0.52	0.44	0.004^**^	0.13	0.42	0.18	0.26
	DOI	0.32	0.04^*^	−0.13	0.41	0.32	0.04^*^	−0.13	0.43	−0.11	0.49
	CPZ	0.12	0.49	0.06	0.74	0.01	0.95	0.04	0.84	0.10	0.57
	IQ	−0.11	0.51	0.02	0.9	−0.26	0.10	0.009	0.96	−0.02	0.90
	SFS	−0.19	0.24	0.10	0.52	0.02	0.92	0.03	0.88	0.10	0.53

Pearson's correlation coefficient is shown except for correlations with symptoms scores in which Spearman's correlation coefficient is displayed. p, significance level; BD, bipolar disorder participants; SZ, schizophrenia participants; BPRS, brief psychiatric rating scale; YMRS, Young Mania rating scale; HRSD, Hamilton rating scale of depression; SAPS and SANS, scale of assessment for positive and negative symptoms, respectively; DOI, duration of illness; CPZ, chlorpromazine daily equivalent; SFS, social functioning scale.

* significant at the unadjusted alpha level = 0.05.

** significant at the adjusted alpha level = 0.01, reflecting correction for 5 multiple comparisons per clinical/demographic variable.

When examining patient groups together, the magnitudes of both motion (Figure 4B) and orientation (Figure 4C) illusions were positively correlated with BPRS such that greater psychiatric symptoms were associated with stronger illusory repulsions (*r*_*s*_ = 0.44, *p* = 0.004; *r*_*s*_ = 0.5, *p* = 0.001, respectively). The same analysis was applied after excluding two potential outliers based on BPRS score (see Figures 4B–C), but still the correlations remained significant (motion: *r* = 0.53, *p* = 0.001; orientation: *r* = 0.47, *p* = 0.002). Similar relationships between motion and orientation tasks and BPRS were found when examining SZ alone (*r*_*s*_ = 0.50, *p* = 0.008; *r*_*s*_ = 0.57, *p* = 0.002, respectively). In BD, the correlation between the orientation illusion and BPRS score was close to significant at the unadjusted alpha level (*r* = 0.48, *p* = 0.06, α = 0.05). Considered together, this pattern of results suggests that psychotic features may play a role in modulating contextual effects of orientation and motion. Illness duration was further associated with stronger orientation and motion repulsion across patient groups (*r* = 0.32, *p* = 0.04 for both) but did not reach significance at the adjusted alpha level (0.01). Finally, performance measures on each and every task were uncorrelated with IQ, CPZ, and social functioning scores (Table 5).

## Discussion

In this study, we examined contextual interactions in bipolar disorder to determine the diagnostic specificity of contextual abnormalities reported in schizophrenia. We (Yang et al., 2013) and others (Dakin et al., 2005; Barch et al., 2012; Tibber et al., 2013) have found that the contextual effect of contrast is weakened in SZ. Yet within the same group of schizophrenia patients, we found the magnitude of contextual modulations associated with luminance, size, orientation, and motion to be similar between SZ and CO (Yang et al., 2013). Tibber et al. (2013) reported similar findings of intact contextual luminance and orientation effects, despite weakened contextual effects of contrast and size (discussed below) in SZ. Utilizing the same contextual tasks from our previous study (Figure 1), here we report relatively normal magnitudes of contextual effects in BD across all features tested, including contrast (Figure 2). With regard to overall contextual modulation strength, the three groups could not be distinguished (Figure 3). Yet, the strength of some contextual illusions covaried with clinical state (Figure 4). In BD, a weaker contrast illusion was associated with greater manic symptoms at the time of testing. We previously reported in SZ that stronger positive and negative symptoms were associated with stronger orientation and motion repulsion illusions. When examining patient groups together, stronger orientation and motion context effects were also associated with greater symptom severity assessed with BPRS. In summary, our findings suggest that the weak contextual modulation of contrast may be a general characteristic of schizophrenia, whereas contextual contrast effects may covary with manic state in bipolar disorder. In addition, the strength of other contextual effects may be modulated by clinical state, especially in schizophrenia. In the following paragraphs, we discuss the implications of our findings with BD and refer readers to our previous study (Yang et al., 2013) for more detailed discussion of visual context processing in SZ.

### Contextual modulation of contrast

In the first study to examine the surround contrast illusion in schizophrenia, Dakin et al. (2005) compared SZ to a healthy control group and a clinical control group that included individuals with affective, personality, and post-traumatic stress disorders. Comparing SZ with such a heterogeneous clinical group cannot probe the diagnostic specificity of the contextual deficit since impairments shared within psychotic spectrum disorders could be washed out by normal performance associated with unrelated illnesses. Thus, we specifically tested an array of contextual interactions specifically in bipolar disorder, an illness that shares many clinical features with schizophrenia (see Introduction). Our study showed that the weakened contextual modulation of contrast is indeed specific to schizophrenia, as BD showed a similar contrast illusion to that of CO (Figures 2D, 3). This is reminiscent of results from a previous study reporting abnormal contrast sensitivity modulation in presence of collinear flanking stimuli in SZ but not in BD (Kéri et al., 2005).

In our study, it is important to note that BD performance on the contrast task could not be distinguished from either that of CO or of SZ. It is possible that certain clinical characteristics were modulating performance within the bipolar group and as a result, some bipolar patients behaved more similarly to schizophrenia patients than control participants. Our findings seem to support this account: Bipolar individuals with greater manic symptoms exhibited a weaker contrast illusion (Figure 4A). Surround contrast modulation is believed to reflect gain control mechanisms in early visual cortical areas (Chubb et al., 1989; Lotto and Purves, 2001). Perhaps, then, hyperdopaminergia related to mania leads to anomalous gain control mechanisms in BD. Indeed, it is known that dopamine mediates processes involved in contrast gain control, particularly in the modulation of visual contrast detection (e.g., Chen et al., 2003).

Given that psychotic symptoms frequently accompany manic phases, another speculation is that the presence of psychotic symptoms allows gain control mechanisms to be modulated by manic phases in BD. Simply put, manic bipolar patients who are prone to psychosis may exhibit weakened gain control mechanisms similar to those of SZ. Such an account would be consistent with early studies reporting impaired backward masking functions in actively psychotic manic patients—similar to impairments in SZ (e.g., Green et al., 1994)—and relatively normal backward masking in non-psychotic hypomanic patients (Saccuzzo and Braff, 1981, 1986). Our study did not have the statistical power to directly compare performance of bipolar patients with (*n* = 9) and without (*n* = 7) a prior history of psychosis. The severity of psychotic symptoms does not appear to modulate the contrast illusion in our pool of bipolar patients, possibly because of the limited range of symptom scores. While similar contextual contrast deficits could be taken as evidence for a shared pathophysiological mechanism between schizophrenia patients and sub-groups of bipolar patients, similar deficits could also manifest from different pathophysiological processes (e.g., Green et al., 1994; further discussion below). Future studies will provide greater insight into this debate by investigating contextual effects along the course of the illness and among different patient sub-groups.

There are at least two caveats in the interpretation of these results. Although BD did not significantly differ in performance on the contrast task from either SZ (*d* = 0.33) or CO (*d* = 0.29), the effect sizes obtained were roughly equivalent to the effect size reported by Barch et al. (2012) who demonstrated a significant group difference between SZ and CO (*d* = 0.31) in their contextual contrast task. Since our data was acquired from a relatively modest sample of bipolar participants, it is possible that given enough subjects the group differences between BD and SZ and between BD and CO would reach significance. Power analysis revealed that approximately 188 participants in each group of BD and CO and 146 participants in each group of BD and SZ would be required to achieve statistically significant group effects for our contextual contrast task, given the obtained effect sizes (power = 0.8, alpha = 0.05).

The second caveat was raised by Barch et al. (2012). In their study, the contextual contrast deficit in SZ was substantially weakened when they excluded individuals who performed poorly on catch trials. Barch and colleagues argued that weakened surround contrast effects in SZ might be attributed to general impairments in attention. Our finding of abnormally weak contrast modulation in SZ is unlikely due to attentional impairments, as the deficit was specific to the contrast task and was not observed in the baseline contrast condition. However, Barch et al.'s results underscore the need for further research into contextual contrast processing in schizophrenia.

### Contextual modulation of orientation and motion

Deficits in motion perception are well established in SZ (review by Chen, 2011) and recent evidence suggests that orientation processing may be disturbed as well (Rokem et al., 2011). Studies have identified abnormal contextual modulation of moving stimuli (Tadin et al., 2006; Chen et al., 2008) and orientation-specific surround suppression (Yoon et al., 2009), although the exact nature of these deficits is still under debate (see Yang et al., 2013). In one study, Chen et al. (2008) reported abnormally strong surround motion repulsion in mildly symptomatic SZ. Consistent with Chen et al.'s results, we previously showed that SZ with stronger motion and orientation repulsion effects also showed greater symptom severity, as assessed with BPRS (Yang et al., 2013). Here, we found that this relationship remained significant when including data from another clinical population—BD—within the psychosis spectrum of disorders (Figures 4B–C). The magnitudes of these repulsion illusions further predicted the severity of a range of clinical symptoms: SZ with greater positive (SAPS) or negative (SANS) symptoms exhibited stronger orientation and motion repulsion effects (Yang et al., 2013) and there was a trend for BD with more severe depressive symptoms (HRSD) to show stronger orientation illusions. Patients with greater duration of illness were more likely to have stronger repulsion effects as well. However, other studies did not find a relationship between clinical symptoms and motion or orientation illusions in SZ and in BD (Chen et al., 2008; Tibber et al., 2013). The discrepancy in results could be attributed to task and stimulus differences or the fact that clinical symptom scores were much higher in these studies (Chen et al., 2008; Tibber et al., 2013). Further investigation will be necessary to ascertain the usefulness of these particular contextual illusions for clinical studies of schizophrenia.

### Contextual modulation of size and brightness

We previously reported relatively normal Ebbinghaus illusion in SZ (Yang et al., 2013). In contrast, Uhlhaas et al. (2004, 2006) reported that both SZ and schizotypal individuals showed a reduced size illusion effect. Notably, this result was observed in only a subset of individuals with disorganization symptoms or thought disorder. Our findings are consistent with Uhlhaas et al.'s in that SZ participants in our study exhibited few, if any, symptoms of disorganization. However, Tibber et al. (2013) reported weakened size illusion among SZ who mostly exhibited few disorganized symptoms. Thus, the relationship between the size illusion and clinical symptoms in SZ is an issue that requires further inquiry.

As far as we know, ours is the first study to examine the role of surrounding context in perceived brightness in bipolar disorder. Our findings show that BD exhibit relatively intact brightness induction. Our group and Tibber et al. (2013) similarly reported normal contextual modulation of luminance in SZ. Taken together, these findings suggest that the early cortical and subcortical mechanisms responsible for the contextual effects in brightness perception (Rossi and Paradiso, 1999; Kinoshita and Komatsu, 2001) may be spared in SZ and in BD.

### Contextual processing: a biomarker for schizophrenia?

There has been a rapid growth in the use of context tasks in clinical trials and large-scale, NIH-supported studies of schizophrenia, particularly tasks focusing on visual context. Given this trajectory, it is imperative to identify the conditions under which contextual processing is compromised in schizophrenia and importantly, to examine the diagnostic specificity of these abnormalities. Recently, we found no clear evidence for a general weakening of contextual visual processing in SZ (Yang et al., 2013), which was later confirmed by Tibber et al. (2013). Using different sets of contextual tasks, both studies reported a weakened contextual contrast effect in SZ. The current study further suggests that the contextual contrast deficit may be specific to SZ, as it was not found in BD (see caveats above). Taken together with previous studies, these findings support the notion that, among different visual context tasks, the contrast illusion may be a more viable candidate for a biomarker of schizophrenia.

The remaining question is whether the contextual contrast deficit is a state- or trait-related characteristic of schizophrenia. The contextual contrast deficit has been reported in both inpatient and outpatient populations and has failed to correlate with any clinical measure at the time of testing (Dakin et al., 2005; Barch et al., 2012; Tibber et al., 2013; Yang et al., 2013). This suggests that the contrast deficit is not influenced by clinical state. Comparison of effect sizes across studies, however, show that the largest effect size was reported in a study of inpatients (Dakin et al., 2005), whereas the smallest effect size was found in a study of outpatients (Barch et al., 2012). Intermediate effect sizes were reported in a smaller cohort of outpatients (Yang et al., 2013) and in a mixture of inpatients and outpatients (Tibber et al., 2013). On this basis, one could speculate that the contrast deficit is indeed modulated by clinical state, as inpatients tend to be actively and severely ill in comparison to clinically stable outpatients. Yet other factors may have contributed to the differences in effect sizes across studies including task differences, medication effects, and sample size (smaller samples tend to enhance effect sizes). Thus, it may be too early to draw conclusions about the role of clinical state in the contextual contrast deficit. This issue would be best addressed with studies examining the contrast illusion along the course of the illness and across a wide range of schizophrenia patients varying in symptom severity.

### Dimensional vs. categorical classification of psychosis spectrum disorders

Converging lines of evidence implicate commonalities between schizophrenia and bipolar disorder, including overlaps in genetic susceptibility, in epidemiologic characteristics, and in neural dysfunction [reviews by Möller (2003) and Maier et al. (2006)]. These findings have revived a long-standing debate as to the relationship between schizophrenia and other psychotic disorders, including bipolar disorder. The traditional dichotomy in the diagnosis of schizophrenia and bipolar disorder has long been challenged by the notion that schizophrenia is not a singular, distinct entity but, instead, forms part of a psychosis continuum (McIntyre, 1949; Craddock and Owen, 2005). However, not all evidence supports a continuous account of psychosis (David, 2010). Abnormalities in neurodevelopment and cognitive function follow distinctly different time courses in the two disorders (Lewandowski et al., 2011). Non-shared genetic risk factors (e.g., Grozeva et al., 2010) and neurobiological distinctions (e.g., structural and functional differences in the brain) also exist between schizophrenia and bipolar disorder (Geuze et al., 2005). Moreover, several empirical findings across multiple domains differentiate SZ from BD. A particularly important example is that of oculomotor control; smooth pursuit eye tracking deficit is a candidate endophenotypic marker for schizophrenia but is intact in bipolar disorder (e.g., Levy et al., 1993; Holzman, 2000; Levy and Sweeney, 2008). Similarly, higher cognitive deficits are severe in schizophrenia but mild or absent in bipolar disorder (Krabbendam et al., 2005) especially with respect to working memory (Park and Holzman, 1992; Pirkola et al., 2005).

Therefore, a singular pathophysiological mechanism is unlikely to account for the two psychotic disorders (Whalley et al., 2012). Similar to the current state of the literature, our findings neither fit perfectly into the continuous or categorical account of psychosis but suggest an alternative approach. The two disorders may be differentiated by their distinct *profiles* of impaired, intact and even enhanced functions. Identifying such profiles across different tasks within a neurobiologically constrained domain may prove to be extremely useful in elucidating the nature of these disorders. Thus, our results emphasize the need for a hybrid model that better captures the complexity in symptoms, deficits, and prognosis within and across diagnostic categories.

Recent initiatives, such as the Research Domain Criteria (RDoC) project, were developed for this purpose (Morris and Cuthbert, 2012). RDoC supports a multi-dimensional approach framed within neuroscience and genomic research to identify core processes underlying clinical features and diagnostic groups. It is too soon to tell whether contextual processing abnormalities contribute to one of those core processes. Future studies should include the investigation of epidemiological characteristics (e.g., risk factors, heritability) to elucidate the role of contextual dysfunction in schizophrenia and psychosis spectrum disorders.

## Summary

Our study systematically measured contextual processing in bipolar disorder and compared those results to equivalent measurements in schizophrenia, to determine the extent to which abnormal contextual interaactions are characteristic of psychosis spectrum disorders in general. We measured contextual effects across a range of visual tasks in individuals with bipolar disorder and compared their performance with that of our previously published findings with schizophrenia and healthy participants tested on those same tasks. Participants with bipolar disorder showed robust contextual effects that were comparable in magnitude to those reported in healthy participants. The contextual contrast illusion, in particular, distinguished performances of bipolar disorder and schizophrenia groups, as individuals with schizophrenia exhibited weakened contrast illusion relative to controls whereas individuals with bipolar disorder did not. Yet, bipolar patients with worse manic symptoms were more likely to have a weaker contrast illusion. Furthermore, the severity of psychiatric symptoms was associated with stronger orientation and motion repulsion illusions, especially in individuals with schizophrenia. These findings may suggest that the pathophysiological mechanisms underlying contextual effects may differ in bipolar disorder compared with schizophrenia.

### Conflict of interest statement

The authors declare that the research was conducted in the absence of any commercial or financial relationships that could be construed as a potential conflict of interest.
